# Telemedicine: Potential impact of the future improved accessibility and waiting list to HGH Sleep Clinics through overbooking strategy, Hamad Medical Corporation, Qatar

**DOI:** 10.5339/qmj.2024.qitc.24

**Published:** 2024-04-08

**Authors:** Abdul-Aziz Al Hashemi, Shaimaa Sherif M. Hassan, Tasleem Raza, Aisha Hussain, Mousa Hussein, Muhannad Salih, Mohamed M A AlMarri, Ibrahim Abdul Rashid, Mansoor Hameed, Zeyad Al Hiyasat, Yasen Ahmed, Hussam Ghali, Ahmed M Mohamoud, Mariam A.O Al-Malaheem, Hisham Abdul Aleem

**Affiliations:** 1 Sleep Lab, Hamad General Hospital, Pulmonary Medicine, Hamad Medical Corporation, Doha, Qatar Email: aalhashemi@hamad.qa; 2 Sleep Disorder Clinic & Lab, HGH, Doha, Qatar; 3 Pulmonary Department, Hamad Medical Corporation, Doha, Qatar; 4 Customer Service Centre for Patient Experience & Staff Engagement, Doha, Qatar; 5 Respiratory Therapy Department, Doha, Qatar; 6 HGH Ambulatory Care Service, Doha, Qatar; 7 Outpatient Department, HGH, Doha, Qatar; 8 Thoracic Surgery Department, Doha, Qatar

**Keywords:** Sleep, Telemedicine, Overbooking, Multidisciplinary, Pulmonary

## Background

The Qatar Ambulatory Pulmonary sleep disorder Clinic is implementing a new approach to managing patient flow by integrating telemedicine follow-up and overbooking strategies^1^ to optimize technology and operational efficiency, ensure timely consultations, and improve patient experience. The hotline service will also be established to optimize clinic efficiency, improve resource utilization, and minimize waiting time for suboptimal diagnostic and therapeutic sleep tests. Telemedicine has become a transformative force in healthcare.^2^

## Methods

**Figure 1.**
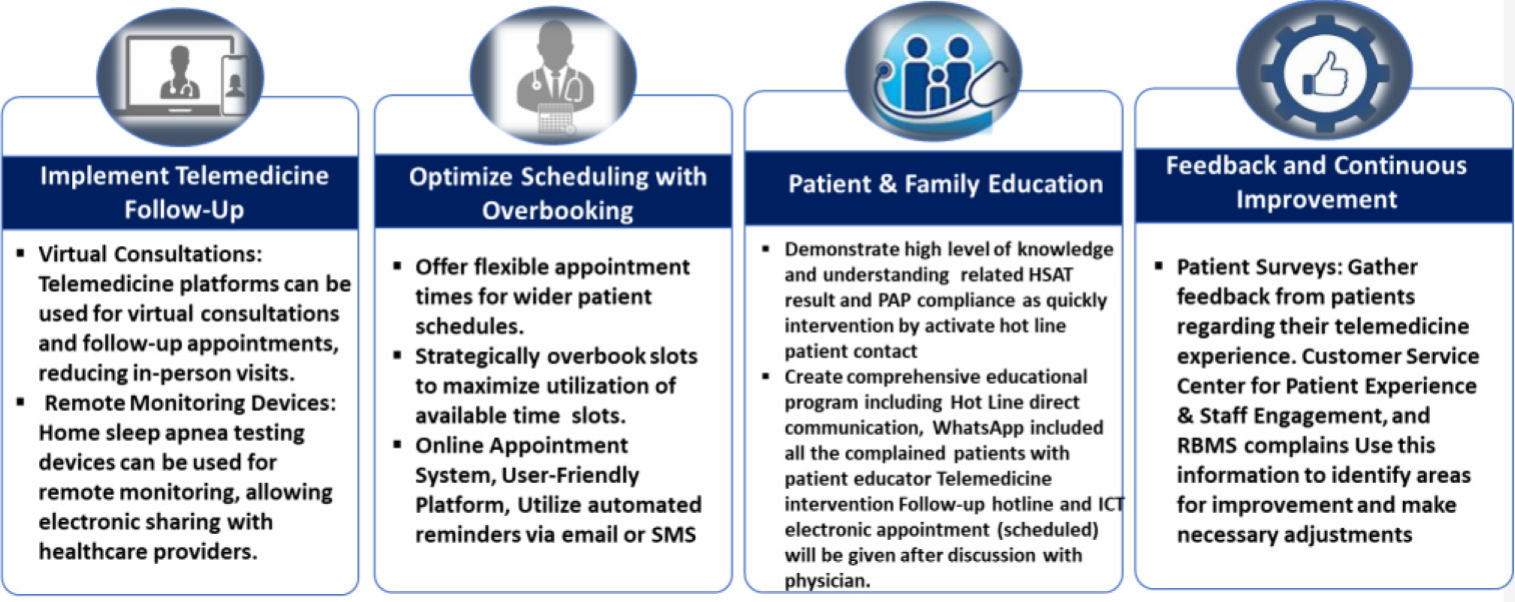
Integration of overbooking strategy and telemedicine follow-up approach.

A multidisciplinary team was formed to review the clinic's current practices and identify barriers to patient flow. A strategy was chosen to integrate overbooking and telemedicine follow-up (Figure 1). Staff education and training were also provided. The team collaborated with HMC NASMAK to evaluate patient feedback and improve the patient experience. They also communicated with clinical informatics team to create encounters, improve electronic communications, and manage appointment systems. Monthly data collection was conducted to evaluate clinical performance and update practice.

## Results/Findings

**Figure 2.**
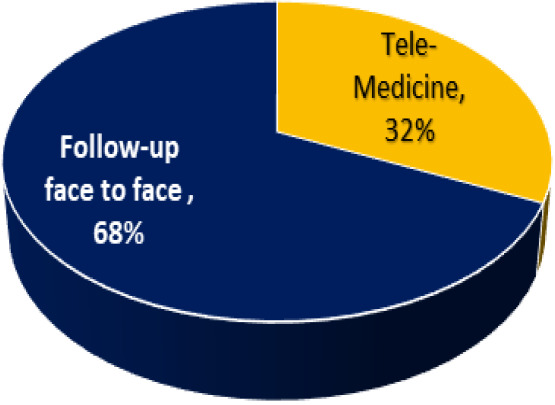
Percentage of patients followed up in pulmonary sleep clinics.

Telemedicine usage is predicted to enhance care quality and reduce healthcare spending by 32%, boosting patient satisfaction, community trust, and teamwork, thereby reducing hospital resource burden and costs (Figure 2).

## Conclusion/Recommendations

The overbooking strategy, combined with telemedicine, can help streamline virtual appointments and ensure efficient use of healthcare professionals' time, ultimately reducing waiting times and improving accessibility for patients seeking sleep-related healthcare services.

## Conflict of Interest

No conflict of interest.

## References

[1] Kichloo A, Albosta M, Dettloff K, Wani F, El-Amir Z, Singh J (2020;). Telemedicine, the current COVID-19 pandemic and the future: A narrative review and perspectives moving forward in the USA. Family Medicine and Community Health.

[2] Masa JF, Rubio M, Findley LJ, Cooperative Group (2000;). Habitually sleepy drivers have a high frequency of automobile crashes associated with respiratory disorders during sleep. American Journal of Respiratory and Critical Care Medicine.

